# Bio-clinical evaluation of collateral score in acute middle cerebral artery occlusion

**DOI:** 10.3906/sag-2103-301

**Published:** 2021-10-23

**Authors:** Esra DEMİR ÜNAL, Hesna BEKTAŞ, Hasan BAYINDIR, Oğuzhan KURŞUN

**Affiliations:** 1 Department of Neurology, Nevşehir City Hospital, Nevşehir, Turkey; 2 Department of Neurology, Faculty of Medicine, Ankara Yıldırım Beyazıt University, Ankara, Turkey; 3 Department of Neurology, Ankara City Hospital, Ankara, Turkey

**Keywords:** Acute ischemic stroke, brain computed tomography angiography, collateral score, lymphocyte monocyte ratio, middle cerebral artery occlusion, RDW

## Abstract

**Background/aim:**

Acute ischemic stroke (AIS) is characterized as a neurological deficit owing to an acute focal damage to the brain by cerebral infarction. A collateral score is the most significant factor evaluating the prognosis of AIS, its relationship with demographic data, serum biochemical parameters, and clinical disability in this field.

**Materials and methods:**

We conducted a single-center retrospective study with 100 patients with AIS within the first 6 h of ischemic stroke. Data for consecutive AIS patients were collected from February 2019 to May 2020. The collateral score was assessed by using developed scoring systems defined by Maas et al. The correlations between collateral score and demographic data, biochemical parameters, NIHSS scores (National Institutes of Health Stroke Scale), mRS (modified Rankin scale) scores were recorded.

**Results:**

The research was performed in 100 patients (median age, 71.55 ± 11.46 years), and there was a statistically significant difference between elevated erythrocyte distribution width (RDW) and Maas collateral score (insular cortex) (p = 0.024) and lymphocyte/monocyte ratio (LMO) and Maas collateral (leptomeningeal) score (p = 0.025).

**Conclusion:**

In patients with acute MCA M1 occlusion, our analysis found a significant association between elevated RDW, low LMO parameters, and collateral score. We assume that, in terms of simple and quick usability in acute stroke prognosis, these parameters are useful and functional as a new biomarker.

## 1. Introduction

Stroke is a medical condition characterized by neurological deficits caused by vascular pathologies and the leading cause of significant disability and death worldwide [1]. The knowledge about stroke has been strengthened by developments in basic science, neuropathology, and neuroimaging. In the ischemic stroke group, which constitutes the vast majority of stroke patients, such as 85%, different classifications are used. The most commonly used classification is the TOAST (Trial of Org 10172 in Acute Stroke) criteria, which is a valid etiological classification system in stroke [2]. TOAST criteria divide patients with ischemic stroke into 5 categories: large artery atherosclerosis, cardioembolism, small vessel occlusion, other causes of stroke, and infarction of undetermined cause. Symptoms and signs vary depending on the size of the lesion in ischemic stroke patients. The size of the lesion depends on which artery is occluded, at what level the artery is occluded, and the anastomosis levels.

The cerebral collaterals are vascular redundancies in the cerebral circulation that can partially maintain blood flow to ischemic tissue when primary conduits are blocked. Anastomoses connecting the distal segments of the MCA to the distal branches of the ACA and PCA (known as leptomeningeal or pial collaterals) after occlusion of the cerebral artery allow partially sustained blood flow in the ischemic penumbra and delay or prevent cell death. The degree of collateral supply is substantial from peripheral leptomeningeal sources and is associated with the existence of smaller amounts of final infarction [3]. Because of their diminutive size and complicated paths, the assessment of collateral supply remains difficult.

The collateral status based on CT angiography (CTA) in the AIS onset is predictive of the outcome [4]. It is readily accessible and delivers a quick review to make choices. CTA enhances the depiction and delineation of infarcts and offers a noninvasive cervical and intracranial circulation assessment [5,6].

In recent years, angiographic collateral system scoring has started to be used more commonly in evaluating stroke prognosis, and a significant association between collateralization can be detected through the angiographic collateral grading system, even a favorable outcome can be achieved [7,8]. The goal of our research is to explore the relationship between collateral score (CS) biochemical parameters and clinical disability in patients with middle cerebral artery (MCA) occlusion admitted to the emergency department with AIS in the first 6 h.

## 2. Materials and Methods

### 2.1. Study design and patient cohort

This is a retrospective, single-center designed study and was conducted between February 2019 and May 2020; 100 AIS patients that were hospitalized with MCA M1 infarction within 6 h were included. Inclusion criteria were as follows: being over the age of 18, admitted to the emergency service within the first 6 h, having middle cerebral artery occlusion in CTA, and no history of another bleeding, malignancy, demyelinating disease, or infectious diseases that can be differentially diagnosed with stroke. AIS type, additional chronic comorbidities, and previously used medicines including antiaggregant or anticoagulant drugs were noted.

Demographic data, admission biochemical parameters (i.e. mean corpuscular volume (MCV), mean corpuscular hemoglobin (MCH), mean corpuscular hemoglobin concentration (MCHC), neutrophil/lymphocyte ratio (NLR), platelet/lymphocyte ratio (PLR), lymphocyte/monocyte ratio (LMR), glucose, urea and creatinine), hospitalization and discharge NIHSS scores (National Institutes of Health Stroke Scale) and discharge mRS (modified Rankin scale) were recorded. The NIHSS scores were classified as minor (0–5), moderate (5–15), severe (15–20), and very severe (20–42), and the mRS score as good (0–1), moderate (2–3), worse (4–5) and exitus ( ≥5). The clinical review board approved this report, and individual patient consent was received from them or relatives.

### 2.2. Scanning protocol

Noncontrast CT (NCCT) and CTA acquisitions were performed according to standard departmental protocols on Revolution CT (GE Healthcare, Illinois, U.S.A) 64 and 128-detector, included pre and postcontrast CT head scans with the following parameters: 120 kilovolts (peak) (kVp), 340 mA, 85 mm/collimation, 1 s/rotation, and table speed of 15 mm/rotation. CTA was performed from the aortic arch to the vertex with the following parameters: 0.7 mL/kg iodinated contrast agent up to a maximum of 90 mL (iohexol, Omnipaque, 300 mg iodine/mL; GE Healthcare, Piscataway, NJ), 5- to 10-s delay, 120 kVp, 270mA, 1 s/rotation, 1.25-mm-thick sections, and table speed of 3.7 mm/rotation. CTA data were automatically processed, including multiplanar 7-mm maximum intensity projection (MIP) reconstructions and 4-mm axial reformats or CTA source images. Image review was independently performed by a clinical neurologist (E.D., H.B., H.B., O.K.) experienced in stroke imaging.

Reviewers were blinded to biological, therapeutic, and imaging follow-up outcomes, but had details about the demographic data and clinical appearance of the cases. Neither of the reviewers had participated in the selection of the patients.

### 2.3. Imaging analysis

#### 2.3.1. Collateral perfusion grading system

The Maas et al.’s method is a 5-point score that compares collateral on the impacted hemisphere with collateral on the unaffected side. The presence of collateral vessels in the sylvian fissure and leptomeningeal convexity area was evaluated based on CTA source images (CTA-SI), and different grades were given for each.

Collateral vessels were graded comparably for the symptomatic hemisphere against the contralateral hemisphere as follows: 1, absent; 2, less than the contralateral normal side; 3, equal to the contralateral normal side; 4, greater than the contralateral normal side; and 5, exuberant. Diminished is defined as Grades 1–2, adequate as Grades 3–5, and augmented as Grades 4–5 (Figures A, B, C, D).

#### 2.3.2. Image processing

The collateral score from the brain CTAs was measured using the Maas System on the images loaded on the computer-loaded PACS (Picture Archiving and Contact System software) in the collateral region in each segment. Poor and bad collateral scores were compared with demographic data, biochemical parameters, and clinical disability scores. In order to decrease bias, all aspects of the study were blinded to clinical knowledge.

### 2.4. Statistical analysis

Calculations were performed by using IBM SPSS Statistics 21.0 (IBM Corp. Released 2012. IBM SPSS Statistics for Windows, Version 21.0. Armonk, NY: IBM Corp.) statistical software package. Results were expressed as the mean SD or median (interquartile range [IQR]) for quantitative variables and as proportions for categorical findings. Frequency analysis of the demographic information of the individuals included in the study was performed, and descriptive statistics of continuous variables such as age were calculated. The average standard deviation and minimum-maximum values of the emergency time and some trading hours were calculated. Frequency analysis of the clinical histories and habits of the individuals involved in the study was also performed. Number and percentage values were calculated. Number and percentage values were given for the information on clinical disability and biochemistry values. Maas collateral scores (for both) were analyzed using the Chi-Square Comparison test to investigate whether there was a statistically significant difference between demographic data, clinical disability, and biochemical parameters. Statistical significance level was accepted as p < 0.05.

## 3. Results

### 3.1. Baseline data

A total of 55% of the individuals in the study were female, and the mean age was 71.55 ± 11.46. The mean time of admission to the emergency department was 1.78 ± 1.00 h, the mean CTA time was 2.61 ± 1.42 h, and the mean blood test time was 2.02 ± 1.11 h.

### 3.2. Clinical assessment

In the study, arrival and discharge NIHSS score and discharge mRS scale were investigated among the parameters of clinical disability. The mean NIHSS score at arrival was 15.18 ± 3.97, the mean NIHSS score at discharge was 10.85 ± 5.18, and the mean mRS at discharge was 3.57 ± 1.52. In the study, 11 of the patients had a stroke in history, and 7 had a stroke in their family history. Fourty-eight of the patients were not smoking (48%). Most of the patients (97.0%) did not have an alcohol habit. A total of 75% of the patients had hypertension and 31% had hyperlipidemia.

### 3.3. Biochemical assessment

Among the serum biochemical parameters, serum hemoglobin, RDW, NLR, LMR, PLR, glucose, urea, and uric acid values were analyzed. Glucose value was low in 1, high in 54 patients; urea value was low in 1, high in 37 patients; the uric acid values were low in 2 and high in 27 patients. Individual hemoglobin levels were low for 27, high for 2 patients; RDW was high for 30 patients; NLR was low for 4, high for 37 patients; PLR was low for 7 and high for 36 patients; LMR was low for 49.9 patients.

### 3.4. Radiologic outcome

The Maas collateral scores of the individuals were analyzed in the insular and leptomeningeal areas using the PACS system in single-phase CT angiography. In the measurement of the Maas collateral score (insular cortex), 24 of them are CS1, 47 of them are CS2, and 29 of them are C3; CS1 in 25, CS2 in 44, CS3 in 23, and CS4 in 8 were identified in the leptomeningeal scores (Table-1). In the classification of the Maas collateral scores, 71 (71%) of the patients had a poor collateral flow in the insular cortex; in the leptomeningeal scores, 69 (69%) of them had a poor collateral flow (Table-2).

### 3.5. Collateral score and clinical disability comparison outcomes

Insular Maas collateral score and clinical disability comparison outcomes are as follows: among the individuals with moderate NIHSS score, 21 had poor collateral flow; among those with severe NIHSS score, 36 had poor collateral flow (Table-3). Leptomeningeal Maas collateral score comparison outcomes are as follows: among the individuals with moderate NIHSS score, 19 had poor collateral flow; among those with severe NIHSS score, 36 had poor collateral flow (Table-4).

### 3.6. Collateral score and biochemical analysis comparison outcomes

Among the people participating in Maas collateral scoring (insular) analysis, 29 of the individuals with normal glucose levels had poor collateral flow, observed in all individuals with low glucose levels; 41 of the individuals with high glucose levels had poor collateral flow. Of the individuals with normal RDW levels, 45 were in the poor collateral flow group, 26 of the individuals with high RDW were in the poor collateral flow group. Maas collateral scoring indicates a statistically significant difference according to RDW levels (p = 0.024). There was no statistically significant difference in Maas collateral scoring (insular) relative to other parameters (i.e. arrival NIHSS score and mRS) except for RDW (p > 0.05) (Table 5).

In the leptomeningeal Maas collateral score analysis, 27 patients with average glucose levels had poor collateral flow, and all individuals with low glucose levels had poor collateral flow (n = 1); 41 of those with elevated glucose levels had poor collateral flow. According to the biochemical parameters evaluated in the analysis, the Maas collateral score (leptomeningeal) did not indicate a statistically significant difference in hemoglobin, RDW, PLO and LMO levels (p > 0.05). Among the individuals in the sample, 30 of those with normal LMR had poor collateral flow; 39 of those with low LMR had poor collateral flow. Maas collateral score (leptomeningeal) indicates a statistically important difference in LMR (p = 0.025) (Table 6). In summary, it was established that Maas collateral score (leptomeningeal) had poor flow in individuals with a low lymphocyte-monocyte ratio.

In summary, a statistically important correlation was observed between the RDW and the Maas collateral score (insular cortex) and the LMR and the Maas collateral score (leptomeningeal) (p < 0.05).

## 4. Discussion

Collateral circulation refers to additional, preexisting vascular pathways that enable the flow of blood to enter the target tissue when the primary canal is blocked [4, 9, 10, 11]. In the brain, the collateral circulation involves venous collaterals and the primary and secondary arterial collaterals [11]. In the case of proximal occlusion or stenosis in these feeding arterial systems, primary arterial collateral refers to short arterial segments in the Circle of Willis that facilitate blood flow between the territories of the internal carotid arteries and the vertebrobasilar system or between cerebral hemispheres [12].

The secondary collateral contains the pial collaterals (also called the leptomeningeal collaterals). Pial collaterals are anastomotic connections based on the pial surface of the cortex, which connect distal branches of the anterior, middle, and posterior cerebral arteries (ACA, MCA, PCA, respectively) [4]. These collateral channels allow blood flow from the territory of an unobstructed artery into the territory of an occluded artery.

Cerebral collateral circulation is a subsidiary vascular network that, after arterial occlusion, is dynamically recruited and can support ischemic regions with residual blood flow [13]. As a result, collateral flow is an effective way to increase blood flow and protect the neurons in the ischemic region. The collateral status assessment can provide useful information on the progression of infarction and the effectiveness of endovascular therapy (EVT) in patients with AIS [13–15]. There are currently several imaging methods for determining collateral flow, including digital subtraction angiography (DSA (criterion standard)) [16], multiphase computed tomography angiography (CTA) [5, 17–19], computed tomography perfusion [17, 20, 21] and magnetic resonance angiography [22]. However, these methods are difficult to implement in the majority of grassroots hospitals because of the need for equipment, the complexity of handling, or other factors. Among the noninvasive approaches for indirect collateral assessment, CTA is the most commonly used for simple usability, comparatively noninvasive, rapid assessment of intra-extra cranial vascular structures [23]. Lima et al. [24] have shown that the grading of leptomeningeal collateral in CTA can be of great aid in finding potential candidates who may benefit from reperfusion therapies and that it is a valid predictor of positive ischemic stroke outcomes. In our research, cranial CTA was used to assess the MCA collateral score for acute stroke patients because of its partially noninvasive nature and ease of use.

Various scoring systems such as Maas system [23], Miteff system [25], modified tan scale [18], Alberta stroke program early CT scoring methodology [5] and Careggi collateral scale [26] have been used to evaluate intracranial CTA collateral in AIS patients. However, there is no consensus about the correct way to assess and rate collateral. Present methods of collateral evaluation are mostly qualitative or semi-quantitative, without any strong evidence of the supremacy of one methodology to another. In our analysis, we used the Maas method to test collateral scores easily and efficiently. The Maas system is a 5-point score system developed by Maas et al. for collateral ratings [27]. This system is based on a comparison of collateral in the affected hemisphere with collateral on the counter-lateral side using the sylvian fissure as an internal control region. Score ranges are 5 (effective), 4 (more on the contralateral side), 3 (equal to those on the contralateral side), 2 (less than on the contralateral side), and 1 (no vascular opacification). Given the predictive clinical effects of reduced or missing collateral vessels, a CTA-based collateral evaluation can provide a clinically useful tool for selecting patients likely to benefit from intra-arterial therapy. Further research that associates the number of collateral vessels, the extent of Perfusion Defect-Infarct Core mismatch, and the clinical outcome can contribute to improvements in patient care [27]. Future studies to investigate these associations would be beneficial.

The pace of ischemic transition is primarily dictated by the consistency and quantity of collateral circulation [28]. Collaterals have recently been analyzed with considerable concern due to their ability to limit the development of the penumbral territory. Various factors have been identified that have an effect on the efficiency and recruiting of certain collateral pathways in AIS [29]. A few of these factors include the age of the patient, the severity of ischemia, congenital variability of primary collateral, and other comorbidities including decreased cardiac output, diffuse cerebral atherosclerosis, tobacco consumption, dehydration, hyperglycemia, urea-uric acid levels, and treatments with an inhibitory effect on elevated blood pressure. In our research, in accordance with the knowledge collected from the epicrisis and laboratory findings of patients, we test modifiable and unmodifiable risk factors with collateral ratings. There was no important association between these demographic risk factors and the collateral score for 100 patients. Hemogram (i.e. MCV, MCH, MCHC, NLR, LMR, PLR) and biochemical values (i.e. glucose, urea, creatinine) were also measured in our sample. We have shown low serum LRO and high RDW to be statistically important.

Increased RDW was found to be correlated with elevated oxidative stress and low antioxidant levels [30, 31]. Oxidative stress can cause damage to RBC membranes and increase fragility. RBCs often minimize the rate of erythroid maturation and erythrocyte life span, which can contribute to an increase in RDW [30]. A strong prediction of mortality has been seen in patients with cardiac failure [32–34], myocardial infarction [35], and peripheral artery disease [36], as well as in the general population [37,38]. Increased RDW is associated not only with death but also with the development of myocardial infarction, stroke, and heart failure in patients with coronary artery disease [30]. Kara et al. compared the RDW values in acute ischemic stroke patients in clusters with different severity scores and found RDW to be a predictive measure of stroke severity [39]. The authors also reported a significant correlation between RDW and other parameters, such as NIHSS and Glasgow coma score (GCS), and found RDW with a cut of point of 14 —which was higher than the current study— with higher sensitivity to differentiate stroke patients from normal subjects (AUC:0.76). In addition, Jia et al. studied 432 patients diagnosed with acute ischemic stroke and confirmed that RDW is closely related to the occurrence of ischemic stroke, revealing the importance of RDW in the progression of an ischemic stroke that may be related to carotid artery occlusion caused by large red blood cells [40]. Also, Turcato et al. indicated that RDW could be used as an independent predictor of stroke severity and prognosis in patients with acute ischemic stroke who underwent antithrombotic therapy [41]. However, whether RDW can predict the incidence of stroke or collateral score remains unclear. Further investigations with a larger sample size are required. In our analysis, a statistically significant difference was observed between increased RDW values and low collateral scores. Contrary to previous studies, there’s no significant correlation found between increased RDW values and clinical disability scores.

Inflammation has been increasingly recognized as a key contributor to acute AIS pathophysiology [42]. Immune system factors are implicated in the initiation and dissemination of ischaemic brain injury and the production of immunosuppression.

Secondary to cerebral ischemia can be linked with intercurrent infections. LMR is a novel biomarker of baseline inflammatory response identified as significant predictors of AIS morbidity and mortality [43–45]. Lux et al. reported that low LMR checked 24 h after thrombosis were independent predictors of a 3-month low functional outcome for acute anterior circulation of large vessel occlusion stroke [46].

Semerano et al. found a significant association of lymphopenia and high NLR values with intracranial hemorrhage in patients with poor collaterals and successful reperfusion [47]. Indeed, there were significant interactions between factors potentially influencing the capacity of leukocytes to reach the ischemic tissue, such as the extent of collateral circulation and the degree of reperfusion, and the course of circulating neutrophil and lymphocyte counts already at admission. However, further experimental and clinical studies of discrete leukocyte subpopulations would be needed to unravel the complex effects of the immune system in the pathophysiology of ischemic stroke and its collateral system effect, as they could assist in the identification of new therapeutic strategies of neuroprotection. There is no published study yet to examine the effect of lymphocyte profile or LMR on the collateral score. In our study, LMR and collateral scores, clinical disability scales, and demographic data were compared. There was a statistically obvious distinction between low LMR and poor collateral scores.

Limitations of the study are given in detail below.

The exclusion criteria restricted the studied population to the people who applied to Ankara City Hospital Emergency Department, as the study setting did not allow for follow-up in other hospitals. This may create a lack of follow-up for stroke survivors of admissions to other hospitals. The present study was based on a detailed interview with the patient (and/or a carer) that was carried out within 24 h of hospital admission for acute stroke. The parameters examined within the scope of the study do not include the significance level of the parameters that have reached statistical significance in subacute or chronic return of stroke. In addition, the limited patient population included in the study brings to mind the idea that different results can be obtained when similar studies are conducted with larger patient groups of different ethnic origins. As stated in the text of our study, we believe that the parameters found statistically significant in terms of acute stroke prognosis will be a guide for new studies to be conducted in this area.

## 5. Conclusion

Our research provides a comparative analysis of the different predictor and unchangeable risk factors and clinical disability scores for intracranial Maas collateral score system for early prognosis in AIS patients. Higher RDW and low LMR amounts are reliable indicators of poor collateral scores. We assume that with more studied efficacious collateral scoring methods, it will become a common application of the acute stroke procedure to assess the prognosis. Further prospective trials, with the assessment of additional knowledge on risk factors, are advised to establish improved scoring criteria for intracranial collateral in AIS. In this regard, our research will encourage clinicians.

## Figures and Tables

**Figure:**
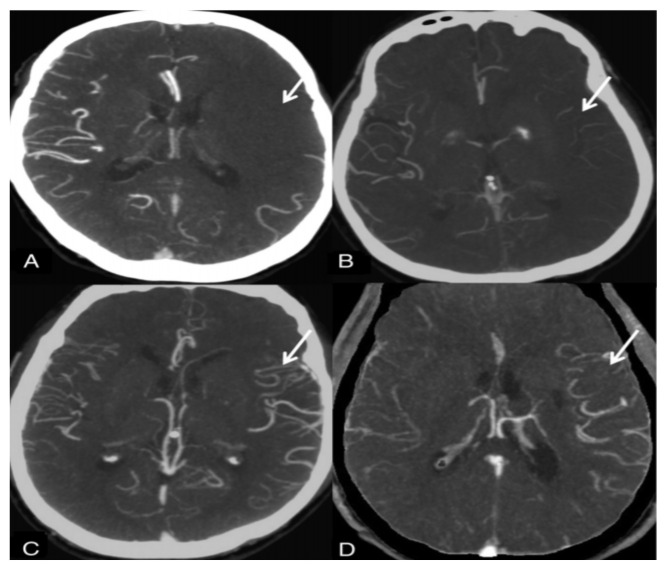
Maas system. A, No vessel opacification. B, Opacification less than that on the contralateral side. Opacification equal to that on the contralateral side is not shown. C, More opacification than that on the contralateral side. D, Exuberant.

**Table 1:** Maas collateral scores distribution.

	n (%)
**MAAS COLLATERAL SCORE (INSULAR)**	
CS1 (Poor Collateral Flow)	24 (24.0)
CS2 (Poor Collateral Flow)	47 (47.0)
CS3 (Good Collateral Flow)	29 (29.0)
**MAAS COLLATERAL SCORE (LEPTOMENINGEAL)**	
CS1 (Poor Collateral Flow)	25 (25.0)
CS2 (Poor Collateral Flow)	44 (44.0)
CS3 (Good Collateral Flow)	23 (23.0)
CS4 (Good Collateral Flow)	8 (8.0)

**Table 2:** Maas collateral scores distribution (grouped).

	n (%)
**MAAS COLLATERAL SCORE (INSULAR)**	
Poor Collateral Flow	71 (71.0)
Good Collateral Flow	29 (29.0)
**MAAS COLLATERAL SCORE (LEPTOMENINGEAL)**	
Poor Collateral Flow	69 (69.0)
Poor Collateral Flow	31 (31.0)

**Table 3:** Comparison of clinical disability and maas collateral scores (insular).

	MAAS COLLATERAL SCORES (INSULAR)		Test Statistics	
Clinical Disability	Poor Collateral Flow n (%)	Good Collateral Flow n (%)	*x* ^2^	p
**Arrival NIHSS Score**				
Moderate (5–14)	21 (65.6)	11 (34.4)	4.326	0.115
Severe (15–19)	36 (67.9)	17 (32.1)		
Very Severe (20–42)	14 (93.3)	1 (6.7)		
**Discharge NIHSS Score**				
Minor (0–4)	4 (50.0)	4 (50.0)	2.518	0.472
Moderate (5–14)	31 (64.6)	17 (35.4)		
Severe (15–19)	15 (78.9)	4 (21.1)		
Very Severe (20–42)	3 (75.0)	1 (25.0)		
**mRS**				
Good (0–1)	0	3 (100.0)	NA	-
Moderate (2–3)	18 (58.1)	13 (41.9)		
Worse (4–5)	34 (77.3)	10 (22.7)		
Exitus (6)	19 (86.4)	3 (13.6)		

* Test statistics cannot be given due to the lack of subjects in the groups.

**Table 4:** Comparison of clinical disability and maas collateral scores (leptomeningeal).

	MAAS COLLATERAL SCORES (LEPTOMENINGEAL)		Test Statistics	
Clinical Disability	Poor Collateral Flow n (%)	Good Collateral Flow n (%)	*x* ^2^	p
**Arrival NIHSS Score**				
Moderate(5–14)	19 (59.4)	13 (40.6)	5.567	0.062
Severe (15–19)	36 (67.9)	17 (32.1)		
Very Severe (20–42)	14 (93.3)	1 (6.7)		
**Discharge NIHSS Score**				
Minor (0–4)	5 (62.5)	3 (37.5)	NA	-
Moderate(5–14)	30 (62.5)	18 (37.5)		
Severe (15–19)	14 (73.7)	5 (26.3)		
Very Severe (20–42)	4 (100.0)	0		
**Mrs**				
	1 (33.3)	2 (66.7)	2.823	0.420
Good (0–1)	20 (64.5)	11 (35.5)		
Moderate (2–3)	31 (70.5)	13 (29.5)		
Worse (4–5)	17 (77.3)	5 (22.7)		
Exitus (6)	19 (59.4)	13 (40.6)		

* Test statistics cannot be given due to the lack of subjects in the groups.

**Table 5:** Comparison of biochemical parameters and maas collateral scores (insular).

	MAAS COLLATERAL SCORES (INSULAR)		Test Statistics	
Biochemical Parameters	Poor Collateral Flow n (%)	Good Collateral Flow n (%)	*x* ^2^	P
**Glucose**				
Normal	29 (64.4)	16 (35.6)	NA	-
Low	1 (100.0)	0		
High	41 (75.9)	13 (24.1)		
**Urea**				
Normal	45 (72.6)	17 (27.4)	NA	-
Low	1 (100.0)	0		
High	25 (67.6)	12 (32.4)		
**Uric Acid**				
Normal	52 (73.2)	19 (26.8)	NA	-
Low	1 (50.0)	0		
High	18 (66.7)	9 (33.3)		
**Creatinin**				
Normal	59 (68.6)	27 (31.4)	NA	-
Low	6 (75.0)	2 (25.0)		
High	6 (100.0)	0		
**Hemoglobin**				
Normal	53 (74.6)	18 (25.4)	1.734	0.420
Low	17 (63.0)	10 (37.0)		
High	1 (50.0)	1 (50.0)		
**RDW**				
Normal	45 (64.3)	25 (35.7)	5.109	0.024
High	26 (86.7)	4 (13.3)		
**Neutrophil Lymphocyte Ratio**				
Normal	39 (66.1)	20 (33.9)	NA	-
Low	4 (100.0)	0		
High	28 (75.7)	9 (24.3)		
**Platelet Lymphocyte Ratio**				
Normal	38 (66.7)	19 (33.3)	2.769	0.250
Low	4 (57.1)	3 (42.9)		
High	29 (80.6)	7 (19.4)		
**Lymphocyte Monocyte Ratio**				
Normal	34 (66.7)	17 (33.3)	0.994	0.330
Low	37 (75.5)	12 (24.5)		

* Test statistics cannot be given due to the lack of subjects in the groups.

**Table 6:** Comparison of biochemical parameters and maas collateral scores (leptomeningeal).

	MAAS COLLATERAL SCORES (LEPTOMENINGEAL)		Test Statistics	
Biochemical Parameters	Poor Collateral Flow n (%)	Good Collateral Flow n (%)	*x* ^2^	P
**Glucose**				
Normal	27 (60.0)	18 (40.0)	NA	-
Low	1 (100.0)	0		
High	41 (75.9)	13 (24.1)		
**Urea**				
Normal	44 (71.0)	18 (29.0)	NA	-
Low	1 (100.0)	0		
High	24 (64.9)	13 (35.1)		
**Uric Acid**				
Normal	51 (71.8)	20 (28.2)	NA	-
Low	2 (100.0)	0		
High	16 (59.3)	11 (40.7)		
**Creatinin**				
Normal	59 (68.6)	27 (31.4)	NA	-
Low	4 (50.0)	4 (50.0)		
High	6 (100.0)	0		
**Hemoglobin**				
Normal	52 (73.2)	19 (26.8)	2.132	0.344
Low	16 (59.3)	11 (40.7)		
High	1 (50.0)	1 (50.0)		
**RDW**				
Normal	47 (67.1)	23 (32.9)	0.376	0.540
High	22 (73.3)	8 (26.7)		
**Neutrophil Lymphocyte Ratio**				
Normal	38 (64.4)	21 (35.6)	1.420	0.492
Low	3 (75.0)	1 (25.0)		
High	28 (75.7)	9 (24.3)		
**Platelet Lymphocyte Ratio**				
Normal	35 (61.4)	22 (38.6)	5.456	0.065
Low	4 (57.1)	3 (42.9)		
High	30 (83.3)	6 (16.7)		
**Lymphocyte Monocyte Ratio**				
Normal	30 (58.8)	21 (41.2)	5.039	0.025
Low	39 (79.6)	10 (20.4)		

* Test statistics cannot be given due to the lack of subjects in the group
